# Successful reperfusion for better outcomes in medium vessel occlusion: Penumbral salvage versus infarct volume reduction

**DOI:** 10.1093/esj/23969873251360492

**Published:** 2026-01-01

**Authors:** Guangchen He, Tingyu Yi, Jiangshan Deng, Liming Wei, Haitao Lu, Xiaohui Lin, Yan Zhang, Guihua Miao, Yueqi Zhu

**Affiliations:** Department of Radiology, Shanghai Sixth People’s Hospital Affiliated to Shanghai Jiao Tong University School of Medicine, Shanghai, China; Department of Interventional Radiology, Fengxian Hospital, The Third School of Clinical Medicine, Southern Medical University, Shanghai, China; Department of Neurointervention, Zhangzhou Affiliated Hospital of Fujian Medical University, Fujian, China; Department of Neurology, Shanghai Sixth People’s Hospital Affiliated to Shanghai Jiao Tong University School of Medicine, Shanghai, China; Department of Radiology, Shanghai Sixth People’s Hospital Affiliated to Shanghai Jiao Tong University School of Medicine, Shanghai, China; Department of Radiology, Shanghai Sixth People’s Hospital Affiliated to Shanghai Jiao Tong University School of Medicine, Shanghai, China; Department of Neurointervention, Zhangzhou Affiliated Hospital of Fujian Medical University, Fujian, China; Department of Neurointervention, Zhangzhou Affiliated Hospital of Fujian Medical University, Fujian, China; Department of Neurology, The First People’s Hospital of Kunshan, Kunshan, Jiangsu Province, China; Department of Neurology, The First People’s Hospital of Kunshan, Kunshan, Jiangsu Province, China; Department of Neurology, The First Affiliated Hospital of Anhui Medical University, Hefei, China; Department of Radiology, Shanghai Sixth People’s Hospital Affiliated to Shanghai Jiao Tong University School of Medicine, Shanghai, China; Department of Interventional Radiology, Fengxian Hospital, The Third School of Clinical Medicine, Southern Medical University, Shanghai, China

**Keywords:** Medium vessel occlusion, reperfusion, penumbra salvaged index, follow-up infarct volume, CT perfusion

## Abstract

**Background:**

The benefits of endovascular thrombectomy (EVT) over medical treatment for medium vessel occlusion (MeVO) remain uncertain. Understanding how vascular reperfusion leads to favorable outcomes is crucial. This study examines whether penumbra salvage and infarct volume reduction quantify EVT benefits in MeVO patients and assesses their impact on clinical improvement post-reperfusion.

**Methods:**

We conducted a multicenter, observational study analyzing MeVO patients who underwent thrombectomy and received multimodal CT imaging from January 2020 to June 2024. EVT efficacy was evaluated by measuring follow-up infarct volume (FIV) on CT scans 24–48 h post-procedure and calculating the penumbra salvage index (PSI). PSI is the ratio of salvaged tissue volume (difference between baseline delay time (DT) >3 s volume and FIV) to baseline DT >3 s volume. Mediation analysis assessed PSI and FIV’s contributions to successful reperfusion and functional outcomes.

**Results:**

Of 338 patients, 241 (72%) achieved successful reperfusion. Median FIV was 21 mL (IQR 12–32 mL), and median PSI was 0.68 (IQR 0.50–0.82). Successful reperfusion was linked to a 0.10 increase in PSI (95% CI: 0.05–0.15, *p* < 0.001) and a 4.36 mL reduction in FIV (95% CI: 1.31–7.20, *p* = 0.005). Successful reperfusion predicted improved outcomes, with an adjusted odds ratio (aOR) of 1.92 (95% CI: 1.08–3.47, *p* = 0.020) for excellent outcomes (modified Rankin Scale (mRS) score 0–1) and an aOR of 1.70 (95% CI: 1.01–2.89, *p* = 0.024) for functional independence (mRS score 0–2). PSI and FIV accounted for 44% and 16%, respectively, of the effect of reperfusion on excellent outcomes.

**Conclusions:**

In acute MeVO patients, penumbra salvage significantly mediates the beneficial relationship between reperfusion and excellent clinical outcomes, more so than infarct volume reduction.

## Graphical Abstract

**Graphical abstract:**
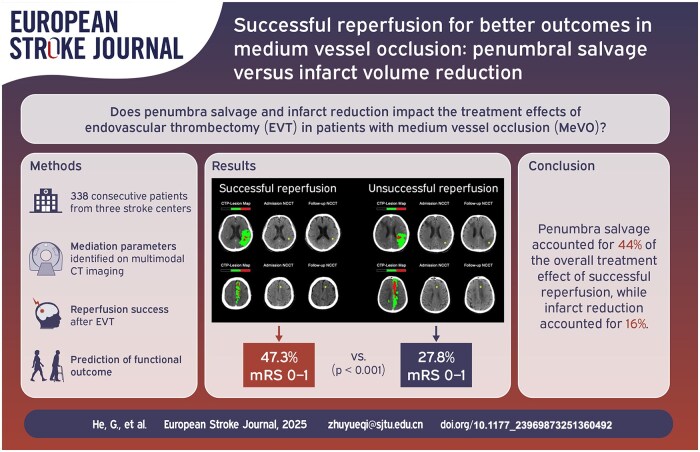

Endovascular thrombectomy (EVT) has become the standard treatment for anterior circulation large vessel occlusions (LVO), including the intracranial internal carotid artery (ICA) and the M1 segment of the middle cerebral artery (MCA) in patients with acute ischemic stroke (AIS) since 2015.^1^ However, patients with more distal occlusions, including medium vessel occlusion (MeVO) in the M2/M3 segments of the MCA or the A2/A3 segments of the anterior cerebral artery (ACA), are frequently excluded. AIS with MeVO typically presents with milder symptoms and a smaller clot burden, which may be more responsive to intravenous thrombolysis.^2,3^ Consequently, the necessity of EVT for MeVO is debated, as its safety and efficacy remain inadequately defined.^4^

Several retrospective, nonrandomized studies have provided preliminary evidence on the safety and efficacy of EVT for MeVO stroke.^5,6^ A post hoc analysis of the HERMES collaboration suggested that EVT might benefit patients with proximal M2 occlusion.^7^ However, two recent randomized controlled trials evaluating EVT for MeVO stroke failed to demonstrate significant improvements in functional outcomes compared to standard medical therapy alone.^8,9^ This lack of benefit may be attributed to several factors, including a relatively lower recanalization rate with EVT, infrequent use of advanced imaging techniques like CT perfusion (CTP), and prolonged workflow times. Additionally, the variability in treatment response for MeVO cases may be attributed to their heterogeneity, which includes posterior P1 or anterior proximal M2 occlusions as well as more distal branch occlusions, such as the P3 and M4 segment. More distal branches, like the M4 segment, are often end arteries that lack collateral circulation, potentially limiting therapeutic efficacy. Therefore, understanding the relationship between vascular recanalization and favorable clinical outcomes following EVT in MeVO cases, and elucidating the underlying mechanisms through quantifiable imaging metrics, is crucial.

We hypothesize that the benefits of EVT in MeVO primarily arise from the salvage and preservation of ischemic brain tissue. To validate this hypothesis, robust imaging biomarkers are required. Follow-up infarct volume (FIV) has emerged as an early measure of treatment efficacy, offering an objective measure of the pathological response to EVT and serving as an early predictor of functional outcome.^10^ However, other studies indicate that FIV accounts for only a small proportion (12%–14%) of the treatment effect.^11,12^ The translation of ischemic tissue injury to functional outcome in MeVO is complex, particularly due to variations in penumbra salvage.^13^ The impact of recanalization in LVO may not directly apply to MeVO, as its smaller tissue territory at risk may result in a reduced benefit. A recently introduced CT-based method, the penumbra salvage index (PSI), quantifies the degree of penumbra rescue, has been proposed as a pathophysiological biomarker.^14^ Higher PSI levels in hypoperfused lesions are associated with favorable pretreatment collateral circulation and increased odds of functional independence.^15,16^ Thus, a higher PSI may help explain the discrepancy between improved outcomes and FIV following EVT, particularly in MeVO, where baseline infarct core on CTP is often limited.

This study aims to evaluate the extent to which the benefit of reperfusion in MeVO on functional outcome is mediated by penumbra salvage and infarct reduction, using quantitative imaging biomarkers. By comparing the relationships between these treatment-related pathophysiological features and successful reperfusion, this study seeks to clarify treatment effects and provide better research strategies and inclusion criteria for future clinical studies on MeVO.

## Materials and methods

### Study population

This retrospective study analyzed anonymized registry data from three high-volume stroke centers (Center 1**, Center 2**, and Center 3**), adhering to local ethical guidelines and the Declaration of Helsinki. This multi-center retrospective study was approved by the hospital institutional review board, and written informed consent was obtained from all participants or legal representatives. Patients presenting within 24 h of anterior circulation MeVOs in the M2/M3 segment of the MCA or the A2/A3 segment of the ACA and undergoing EVT between January 2020 and June 2024 were screened using the following inclusion criteria: (1) multimodal CT with non-contrast CT (NCCT) and CTP before reperfusion therapy; (2) initial Alberta Stroke Program Early CT Score (ASPECTS) ⩾6 on admission NCCT; (3) admission National Institutes of Health Stroke Scale (NIHSS) score ⩾6 or 3–5 with disabling symptoms (e.g. hemianopia, aphasia, loss of hand function); (4) follow-up CT within 24–48 h; (5) absence of severe motion artifacts. EVT was performed via a femoral artery approach under general anesthesia or conscious sedation. The thrombectomy procedures were standardized across centers, using clot retrieval or direct aspiration. Device choice was at the operator’s discretion. Reperfusion status was classified at both the initial and final DSA series following thrombectomy according to the extended Thrombolysis in Cerebral Infarction (eTICI) Scale score: (1) successful reperfusion (eTICI 2b–3); (2) unsuccessful reperfusion (persistent MeVO, eTICI 0–2a).^17,18^

Data on demographics, vascular risk factors, NIHSS score, use of intravenous recombinant tissue-type plasminogen activator (rtPA), time metrics, and procedural variables were collected from stroke database. All patients had 90-day modified Rankin Scale (mRS) scores recorded by neurologically trained personnel blinded to CT imaging and angiographic results. Functional outcome was defined as mRS score of 0–1 (excellent) and mRS score of 0–2 (independent).

### Imaging acquisition

Upon admission, patients underwent a comprehensive stroke imaging protocol, including NCCT and CTP on 128-, 256- or 640-slice CT scanners (Philips iCT 256, Siemens Somatom Definition Force, United Imaging Healthcare uCT960+). NCCT parameters: 120 kV, 280–340 mA, 5.0 mm slice reconstruction, and 1 mm increments. CTP parameters: 100 kV, 200–250 mA, 5-mm slice reconstruction, 1.50 s slice sampling rate (minimum 1.33 s), 45–65 s scan time, and biphasic injection of 30–50 mL iodinated contrast at 4–6 mL/s, followed by 30 mL saline. Datasets with severe motion artifacts were excluded.

### Imaging analysis

We have refined our vascular classification on CTA or DSA by specifying the counts for M2 and M3 segments and further subdividing the M2 segment into proximal and distal parts. The proximal M2 segment extended from the MCA bifurcation/trifurcation to 1 cm distally.^19^ The distal M2 segment spans from this point to the circular sulcus of the insula, while M3 denoted the opercular segment.

CTP data were processed using MIStar software (Apollo Medical Imaging Technology, Melbourne, Australia). Parameters were calculated using a singular value decomposition algorithm with delay and dispersion correction.^20^ Acute hypoperfused volume was defined as delay time (DT) >3 s, severe hypoperfused volume as DT >6 s, and ischemic core infarction as relative cerebral blood flow (rCBF) <30% of the contralateral hemisphere. Penumbral tissue was the difference between hypoperfused lesion and ischemic core. The collateral index was calculated as the ratio of DT >2 s hypoperfusion volume to DT >6 s hypoperfusion volume, with higher ratios indicating more delayed flow arrival.^21^ Follow-up infarct volume and symptomatic intracranial hemorrhage (sICH) were evaluated on non-contrast NCCT at 24–48 h post-admission, according to the ECASS-II criteria.^22^ Patients with significant parenchymal hemorrhagic transformation (PH2) on follow-up imaging were excluded. Infarct volume was delineated slice by slice by an experienced, board-certified neuroradiologist using the paintbrush mode in the ITK-SNAP software (http://www.itksnap.org), blinded to clinical and angiographic data. The delineated volumes were reviewed by an independent neuroradiologist for verification. The penumbra salvage index (PSI) was calculated as the ratio of salvaged tissue volume (i.e. the difference between DT >3 s volume at baseline and FIV) to DT >3 s volume at baseline.

### Statistical analysis

Data were summarized as means with standard deviation (SD), medians, and interquartile ranges (IQR) or number and frequencies, as appropriate. The Kolmogorov–Smirnov test was used to assess normality. Between-group comparisons for categorical variables were performed using the χ^2^ test, and for continuous variables using Student’s *t*-test or the Mann–Whitney *U* test, depending on data distribution. Logistic regression analysis was used to identify independent predictors of functional outcome (mRS 0–1 and mRS 0–2). Variables with *p* < 0.1 in univariable analysis were included in the final multivariable model. Similarly, linear regression analysis was performed to identify independent predictors of PSI and FIV, using backward variable selection. Age, baseline NIHSS, occlusion site, ischemic core volume, and recanalization status were included in the model.

Mediation analysis was conducted using the Baron and Kenny’s template and Vanderweele’s method^23^ to evaluate how much penumbra salvage and infarct volume explain the functional outcome at 90 days after successful reperfusion. Three pathways were tested: (a) the association between successful reperfusion and excellent outcome; (b) the association between successful reperfusion and PSI/FIV; (c) the association between PSI/FIV and excellent outcome. Mediation (indirect effect) was estimated after confirming these associations through linear regression analysis. PSI and FIV were log-transformed (log + 1) to satisfy model assumptions of normality and homoscedasticity. A Bonferroni correction was applied to adjust for multiple comparisons. Finally, subgroup analysis was performed to examine the mediation effects of PSI and FIV in patients with complete reperfusion (eTICI 2c/3). All statistical analyses were performed using R software (version 4.0.3; R Foundation for Statistical Computing). A two-sided *p* < 0.05 was considered statistically significant.

## Results

### Patients characteristics

**Figure 1.**
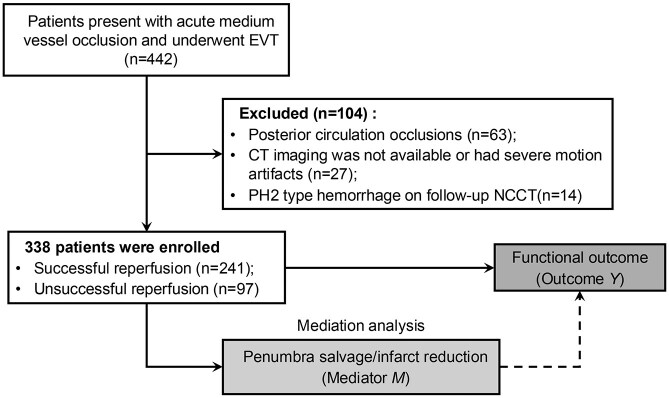
Flow diagram illustrating the patient inclusion process in this study. EVT: endovascular thrombectomy; NCCT: non-contrast CT; PH2: parenchymal hematoma 2.

Of 442 patients with acute MeVO and received EVT across three centers, 338 met eligibility criteria and were included in the analysis (Figure 1). The median age was 73 years (interquartile range (IQR) 66–81 years), with 192 (57%) being males. At baseline, the median NIHSS was 10 (IQR 7–14) and the median ASPECTS was 10 (IQR 8–10). The median ischemic core and penumbra volumes were 4 mL (IQR 2–8 mL) and 57 mL (IQR 40–85 mL), respectively, as summarized in Table 1. Successful reperfusion was achieved in 241 (72%) patients, while 97 (28%) had unsuccessful reperfusion. The median time from puncture to reperfusion was significantly shorter in successful reperfusion cases (median 50 min, IQR 35–67 min) compared to unsuccessful ones (median 63 min, IQR 42–88 min, *p* < 0.001).

**Table 1.** Patient characteristics.

Characteristic	Total (*n* = 338)	Successful reperfusion (*n* = 241)	Unsuccessful reperfusion (*n* = 97)	*p* value
Age (years), median (IQR)	73 (66–81)	73 (66–81)	74 (66–83)	0.948
Male, *n* (%)	192 (57)	136 (56)	56 (58)	0.903
Clinical history, *n* (%)				
Diabetes mellitus	78 (23)	51 (21)	27 (28)	0.201
Hypertension	171 (51)	125 (52)	46 (47)	0.473
Atrial fibrillation	116 (34)	87 (36)	29 (30)	0.312
Coronary artery disease	49 (14)	34 (14)	15 (15)	0.735
Dyslipidemia	61 (18)	41 (17)	20 (21)	0.438
Current smoking	52 (15)	35 (15)	17 (18)	0.507
Baseline NIHSS, median (IQR)	10 (7–14)	10 (7–13)	10 (7–14)	0.594
IVT, *n* (%)	68 (20)	47 (20)	21 (22)	0.655
Time form last known well to imaging acquisition (min), median (IQR)	300 (156–594)	300 (150–650)	296 (158–540)	0.611
ASPECTS score, median (IQR)	10 (8–10)	10 (8–10)	10 (8–10)	0.970
Site of occlusion, *n* (%)				0.210
Proximal M2 MCA	95 (37)	71 (29)	24 (25)	
Distal M2 MCA	74 (29)	57 (24)	17 (17)	
M3 MCA	87 (34)	55 (23)	32 (33)	
A2/A3 ACA	82 (24)	58 (24)	24 (25)	
Stroke subtypes				0.496
Cardioembolic	171 (51)	119 (49)	52 (54)	
Large artery atherosclerosis	62 (18)	48 (20)	14 (14)	
Cryptogenic	105 (31)	74 (31)	31 (32)	
Ischemic core volume (mL), median (IQR)	4 (2–8)	4 (2–8)	4 (2–8)	0.779
Penumbra volume (mL), median (IQR)	57 (40–85)	58 (42–90)	54 (39–74)	0.188
Collateral index, median (IQR)	0.34 (0.24–0.45)	0.34 (0.25–0.45)	0.33 (0.24–0.45)	0.466
Technique				0.496
Contact aspiration	55 (16)	40 (17)	15 (15)	
Stent-retriever	73 (22)	48 (20)	25 (26)	
Combined technique	210 (62)	153 (63)	57 (59)	
Time form puncture to reperfusion/end of the procedure (min), median (IQR)	51 (36–74)	50 (35–67)	63 (42–88)	<0.001
Time form baseline CT imaging to follow-up noncontrast CT (h), median (IQR)	37 (30–42)	37 (30–42)	36 (31–43)	0.545
Follow-up infarct volume (mL), median (IQR)	21 (12–32)	19 (12–31)	25 (13–33)	<0.001
Penumbra salvage index, median (IQR)	0.68 (0.50–0.82)	0.70 (0.58–0.83)	0.60 (0.43–0.72)	<0.001
Hemorrhagic transformation				0.742
HI 1	52 (15)	40 (17)	12 (12)	
HI 2	30 (8.9)	21 (8.7)	9 (9.3)	
PH 1	14 (4.1)	11 (4.6)	3 (3.1)	
sICH, *n* (%)	24 (7.1)	18 (7.5)	6 (6.2)	0.817
mRS at 90 days, median (IQR)	2 (1–3)	2 (1–3)	3 (1–4)	0.001
mRS ⩽1 at 90 days, *n* (%)	141 (42)	114 (47)	27 (28)	0.001
mRS ⩽2 at 90 days, *n* (%)	199 (59)	154 (64)	45 (46)	0.003

ASPECTS: Alberta Stroke Program Early CT Score; IQR: interquartile ranges; HI: hemorrhagic infarction; IVT: intravenous thrombolysis; MCA: middle cerebral artery; mRS: modified Rankin Scale; mTICI: modified thrombolysis in cerebral infarction; NIHSS: National Institutes of Health Stroke Score; PH: parenchymal hemorrhage; sICH: symptomatic intracerebral hemorrhage.

### Penumbra salvage and infarct reduction

**Figure 2.**
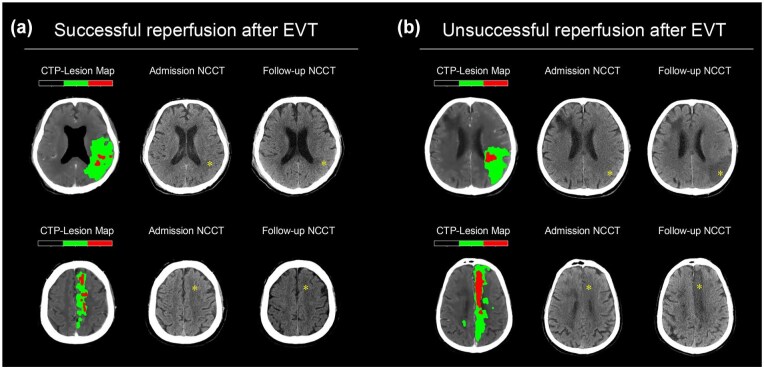
Illustrations of successful and unsuccessful reperfusion following endovascular thrombectomy for medium vessel occlusion. Each case includes lesion maps derived from admission CTP, admission NCCT, and follow-up NCCT. (a) Successful reperfusion is observed in a patient with M2 occlusion (top) with a PSI of 0.93 and an FIV of 5 mL, and in a patient with A2 occlusion (bottom) with a PSI of 0.93 and an FIV of 4 mL. (b) Unsuccessful reperfusion is apparent in a patient with M2 occlusion (top) with a PSI of 0.11 and an FIV of 63 mL, and in a patient with A2 occlusion (bottom) with a PSI of 0.17 and FIV of 65 mL. CTP: CT perfusion; FIV: follow-up infarct volume; NCCT: noncontrast CT; PSI: penumbra salvage index.

Upon imaging follow-up, the median FIV was 21 mL (IQR 12–32 mL), and the median PSI was 0.68 (IQR 0.50–0.82). Multivariate linear regression showed that successful reperfusion was independently associated with higher PSI (coefficient: 0.10, 95% CI: 0.05–0.15, *p* < 0.001) and lower FIV (coefficient 4.36 mL, 95% CI: 1.31–7.20 mL, *p* = 0.005), after adjusting for age, baseline NIHSS, MCA occlusion, and baseline ischemic core volume (Figure 2). Additionally, baseline ischemic core volume was significantly associated with FIV (coefficient: 0.50 mL, 95% CI: 0.14–0.86 mL, *p* = 0.007; Supplementary Table 1).

### Clinical outcomes

**Figure 3.**
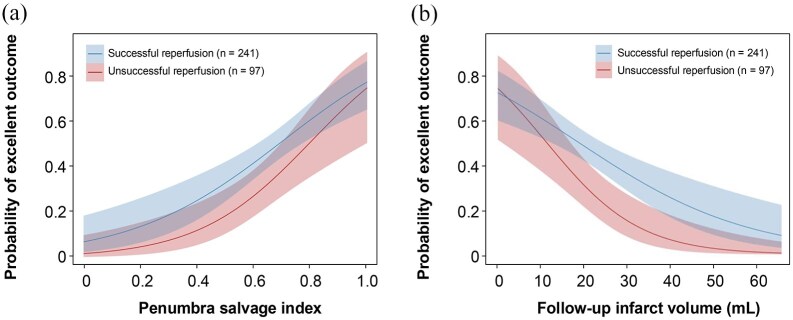
Correlation between successful reperfusion and the penumbra salvage index (a), as well as follow-up infarct volume (b), in relation to the probability of achieving an excellent clinical outcome.

At 90-day follow-up, the median mRS was 2 (IQR: 1–3). An excellent outcome (mRS 0–1) was achieved by 141 (42%) patients, while functional independence (mRS 0–2) was achieved by 201 (59%; Figure 3). Multivariate logistic regression revealed that successful reperfusion was independently associated with excellent outcome (adjusted odds ratio (aOR) 1.92, 95% CI: 1.08–3.47, *p* = 0.020) and functional independence (aOR 1.70, 95% CI: 1.01–2.89, *p* = 0.024). PSI (aOR 1.36, 95% CI: 1.14–1.64, *p* < 0.001) and FIV (aOR: 0.97, 95% CI: 0.94–0.99, *p* = 0.010) were significant independent predictors of excellent outcomes but not functional independence after adjustment for confounders (Table 2).

**Table 2.** Outcome model multivariate logistic regression analysis.

Factors	Excellent outcome (mRS 0–1)		Functional independence (mRS 0–2)	
	Odds ratio (95% CI)	*p* value	Odds ratio (95% CI)	*p* value
Age (years)	0.98 (0.91–1.01)	0.113	0.98 (0.91–1.00)	0.048
Baseline NIHSS	0.94 (0.89–0.99)	0.028	0.94 (0.89–0.99)	0.022
MCA occlusion	0.65 (0.90–1.03)	0.137	0.64 (0.90–1.03)	0.092
Baseline ischemic core volume (mL)	0.97 (0.90–1.03)	0.342	0.98 (0.92–1.04)	0.508
Successful reperfusion	1.92 (1.08–3.47)	0.020	1.70 (1.01–2.89)	0.024
PSI (per 10%)	1.36 (1.14–1.64)	<0.001	1.14 (0.98–1.33)	0.081
Follow-up infarct volume (mL)	0.97 (0.94–0.99)	0.010	0.98 (0.95–1.00)	0.047

CI: confidence interval; FIV: follow-up infarct volume; MCA: middle cerebral artery; mRS: modified Rankin Scale; NIHSS: National Institutes of Health Stroke Score; PSI: penumbra salvage index.

### Mediation of successful reperfusion effect on outcome by PSI and FIV

**Figure 4.**
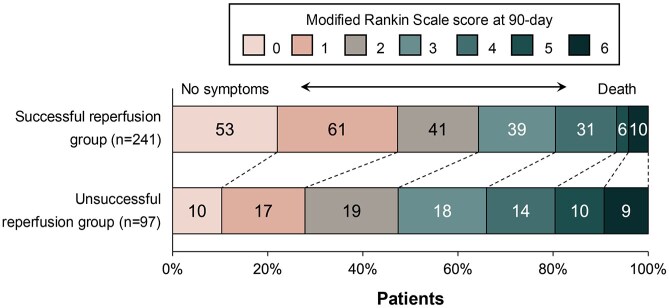
Distribution of modified Rankin Scale score at 90 days post-procedure, categorized by reperfusion status.

Mediation analysis demonstrated that PSI mediated 44% of the effect between successful reperfusion and excellent outcome (total effect: 0.20, 95% CI: 0.90–0.31, *p* = 0.002). The indirect effect mediated by PSI was 0.09 (95% CI: 0.05–0.14, *p* < 0.001). When utilizing FIV as a mediator, the reduction in FIV accounted for only 16% of the effect (total effect: 0.20, 95% CI: 0.08–0.31, *p* = 0.002), with an indirect effect of 0.03 (95% CI: 0.01–0.07, *p* = 0.036; Table 3). Figure 4 illustrates a positive association between successful reperfusion and an increased likelihood of excellent outcome with rising PSI levels. Conversely, an increased FIV correlates with a decreased probability of excellent outcomes in unsuccessful reperfusion cases. However, the indirect effect in the mediation analysis for functional independence did not reach statistical significance.

**Table 3.** Mediation analysis for excellent outcome.

Characteristic	Mediator: penumbra salvage index	*p* value	Mediator: follow-up infarct volume	*p* value
	Estimate (95% CI)		Estimate (95% CI)	
Total effect	0.20 (0.90–0.31)	0.002	0.20 (0.08–0.31)	0.002
ADE	0.11 (0.01–0.22)	0.036	0.17 (0.06–0.27)	0.004
ACME	0.09 (0.05–0.14)	<0.001	0.03 (0.01–0.07)	0.036
Proportion mediated	0.44 (0.22–0.97)	0.002	0.16 (0.01–0.41)	0.036

ACME: average causal mediation effect; ADE: average direct effect; CI: confidence interval.

### Subgroup analysis of reperfusion levels on outcomes

Among patients with successful reperfusion, 76 (32%) achieved eTICI 2b, whereas 165 (68%) reached eTICI 2c/3. Reperfusion with eTICI 2c/3 was independently associated with higher PSI (coefficient 0.12, 95% CI: 0.07–0.17, *p* < 0.001) and lower FIV (coefficient −4.12 mL, 95% CI: 0.99–7.35 mL, *p* = 0.010), adjusted for confounders (Supplementary Table 2). Additionally, eTICI 2c/3 was associated with a higher proportion of excellent outcome (52% vs 28%, *p* = 0.037) and functional independence (68% vs 54%, *p* = 0.031). However, multivariate logistic regression did not find eTICI 2c/3 reperfusion significantly associated with excellent outcome (aOR 1.31, 95% CI: 0.70–2.46, *p* = 0.400) or functional independence (aOR 1.66, 95% CI: 0.89–3.09, *p* = 0.112; Supplementary Table 3). Furthermore, a multivariate logistic regression analysis that included patients with PH2 in the overall population demonstrated that the therapeutic benefits of reperfusion remain significant, even when PH2 patients are considered (Supplementary Table 4).

## Discussion

This study demonstrated that successful reperfusion significantly enhances penumbra salvage, reduces infarct size, and improves functional outcomes in patients with MeVO. Both the PSI and FIV were associated with excellent outcomes. Mediation analysis revealed that PSI and FIV significantly mediated the benefits of reperfusion, accounting for 44% and 16% of the total treatment effect, respectively. Subgroup analysis indicated that eTICI 2c/3 reperfusion was associated with higher PSI and lower FIV; however, it did not significantly impact excellent outcomes or functional independence compared to eTICI 2b reperfusion.

EVT aims to salvage metabolically viable brain tissue by rapidly restoring blood flow. Patients with mild early infarction signs, indicated by MeVO on CTA and/or low hypoperfusion volume on CTP, are often excluded from EVT due to insufficient evidence of benefit and concerns about thrombectomy complications.^24^ Recent clinical trials, including DISTAL and ESCAPE-MeVO, found no significant outcome differences for EVT compared to medical management alone in MeVO cases. The underlying mechanisms for the benefits of reperfusion in MeVO remain inadequately defined, and it is still unclear whether stabilizing the core infarct or salvaging the penumbra is more crucial for patient outcomes. The ESCAPE-MeVO study confirmed that a core and penumbra mismatch was sufficient for inclusion, while the DISTAL study provided more specific imaging criteria within the 6–24 h window.^8,9^ These criteria included a hypoperfusion-hypodensity mismatch on multimodal CT and a diffusion-hyperintensity mismatch on multimodal MR. However, these relatively broad imaging inclusion criteria, while potentially rescuing more patients, may decrease the likelihood of achieving positive clinical outcomes from EVT. The DISTAL and ESCAPE-MeVO study reported successful reperfusion rates of 71.7% and 75.1%, respectively. Given that MeVO thrombectomy does not achieve higher reperfusion rates compared to LVO, cases of unsuccessful reperfusion may significantly impact clinical prognosis. Similarly, our findings indicate that successful reperfusion enhances EVT outcomes, improving both functional independence and excellent outcomes.

Our study extends the understanding of the impact of EVT on clinical outcome by quantitatively assessing penumbra salvage and infarct reduction. Previous studies suggested a minor role for infarct volume reduction (12%–14% of treatment effect), whereas our findings indicate that while infarct reduction is similar, penumbra salvage plays a more significant role in MeVO (44%).^11,12^ This discrepancy may be attributed to the distinct pathophysiology of MeVO, characterized by a smaller ischemic core and a larger penumbra area, allowing for greater tissue salvage. Our mediation analysis highlights the importance of PSI in achieving excellent outcomes, suggesting that salvaging ischemic tissue offers more clinical benefit than merely reducing infarct size. Consequently, strategies to enhance reperfusion and preserve penumbral tissue should be prioritized, including optimizing EVT timing and employing adjuvant therapies. Quantitative imaging biomarkers like PSI can guide decision-making, enabling precise treatment evaluation.

In this study, we used CTP imaging to identify MeVO and estimate ischemic core and hypoperfusion volumes due to its high diagnostic sensitivity, availability, and processing consistency with MIStar software.^25^ However, CTP may overestimate irreversible tissue damage by more than 20% in certain cases, particularly when relying on rCBF-derived thresholds.^26^ This overestimation could be especially pronounced in MeVO patients, especially in distal branches of end arteries with limited or absent collateral circulation. Additionally, irreversible ischemic changes that are evident on NCCT may not be identified as part of the ischemic core on CTP. This discrepancy can result in a larger infarct growth than initially measured.^27^ To address these concerns, we employed final infarct volume as a mediating factor in this study, as it offers a more accurate estimate of ischemic injury when assessing the effects of EVT in MeVO.

Incomplete reperfusion may substantially increase infarct volume, compromising the accuracy of PSI assessments and adversely impacting long-term functional outcomes.^28^ In our subgroup analysis, we found that eTICI 2c/3 reperfusion was significantly associated with higher PSI (*p* < 0.001) and lower FIV (*p* = 0.010), confirming that greater reperfusion rescues more ischemic tissue. However, we found no significant differences in excellent outcomes or functional independence between patients achieving eTICI 2b and those with eTICI 2c/3 reperfusion. This equipoise may reflect heterogeneous collateral circulation in MeVO patients, which could mitigate the incremental benefits of higher reperfusion grades.^29^ These observations suggest that higher grades of reperfusion may not substantially improve outcomes in patients with well-developed collaterals.^30^ Furthermore, the mRS may lack the sensitivity required to discern differences in 90-day outcomes between moderate and complete reperfusion in MeVO patients. One possible explanation for this is the generally low level of physical disability observed, even among patients who didn’t undergo successful EVT, as evidenced by a median 90-day mRS score of 2. This finding is also highly consistent with the results published in the DISTAL and ESCAPE-MeVO studies. Overall, these findings suggest that striving for higher reperfusion grades may not improve functional outcomes in MeVO patients based on our retrospective data. Therefore, interventionalists should carefully evaluate the risk-benefit profile of additional maneuvers once eTICI 2b reperfusion is achieved in EVT.

Our results highlight the need for further prospective studies on EVT for MeVO, particularly given the safety concerns related to smaller and more tortuous distal vessels. In our study, the rate of sICH was 7.1%, consistent with previous studies.^31,32^ Despite similar adverse event risks to LVO patients, successful reperfusion has been linked to symptomatic hemorrhage, influenced by baseline ischemic changes and procedural factors.^6^ Future studies with larger cohorts are needed to clarify the role of successful reperfusion in mediating sICH risk.

### Limitations

The study has several limitations, including its retrospective design, lack of randomization, and potential selection bias. Our study defined M2 occlusions solely by morphological vascular anatomy. However, occlusions affecting the dominant M2 branch – which may produce clinical symptoms functionally equivalent to M1 occlusions (functional M1) – could confound quantitative analyses of MeVO outcomes. Further, this study was conducted in China, where the higher prevalence of intracranial atherosclerosis may limit the generalizability of findings to other populations. The accuracy of eTICI grading for reperfusion in MeVO may be inferior to that in LVO. Exclusion of patients with type 2 parenchymal hemorrhage may also affect results. Final infarct volumes measured on NCCT are less precise than diffusion-weighted MRI. Meanwhile, a delay between baseline imaging and vessel recanalization, along with thrombus migration or distal embolization, may increase FIV and decreased PSI, thereby diminishing the effectiveness of EVT. Additionally, the mediation effect of penumbra salvage is limited by MIStar software use, although its consistency with other software like RAPID has been demonstrated.^33^ Despite these limitations, the study’s strengths include a multicenter design, standardized imaging protocols, and rigorous statistical analysis. Future studies should explore additional mediators, such as edema reduction, neuroinflammation, and tissue reperfusion quality to identify novel therapeutic targets for enhancing reperfusion outcomes in MeVO.^34,35^

## Conclusion

Endovascular recanalization significantly enhances penumbra salvage and reduced infarct size in patients who received EVT with an anterior circulation acute ischemic stroke and a MeVO. Penumbra salvage accounted for 44% of the overall treatment effect of successful reperfusion, while infarct reduction accounted for 16%. This study offers valuable insights into infarction pathophysiology and supports the use of quantitative imaging biomarkers for decision-making in MeVO management. Further prospective studies with larger sample sizes are needed to validate these findings.

## Supplementary Material

sj-docx-1-eso_23969873251360492

## Data Availability

This study’s data are available from the corresponding author upon reasonable request.
