# Supplementary material for: Successful reperfusion for better outcomes in medium vessel occlusion: Penumbral salvage versus infarct volume reduction
Source: Eur Stroke J. 2026 Jan 1;11(1):23969873251360492. doi: 10.1093/esj/23969873251360492 (PMC12866253; doi:10.1093/esj/23969873251360492)
Supplement: sj-docx-1-eso_23969873251360492 [file sj-docx-1-eso_23969873251360492.docx]

**SUPPLEMENTARY MATERIAL**

| **Supplementary Table 1: Mediator models multivariate linear regression analysis** | | | | |
| --- | --- | --- | --- | --- |
|  | **Mediator model 1**  ***M*: Penumbra salvage index**  **Coefficient (95% CI)** | ***P* Value** | **Mediator model 2**  ***M*: Follow-up infarct volume**  **Coefficient (95% CI)** | ***P* Value** |
| Age | -0.01 (-0.01, 0.01) | 0.443 | -0.05 (-0.16, 0.07) | 0.446 |
| Baseline NIHSS | -0.01 (-0.01, 0.01) | 0.296 | 0.33 (0.02, 0.63) | 0.035 |
| MCA occlusion | -0.01 (-0.04, 0.02) | 0.574 | 0.46 (-2.69, 3.61) | 0.774 |
| Baseline Ischemic core volume | -0.01 (-0.01, 0.01) | 0.962 | 0.50 (0.14, 0.86) | 0.007 |
| Successful reperfusion | 0.10 (0.05, 0.15) | <0.001 | -4.36 (-7.20, -1.31) | 0.005 |
| CI refers to confidence interval; MCA, middle cerebral artery | | | | |

| **Supplementary Table 2: Outcome model multivariate logistic regression analysis in the overall population (including patients with parenchymal hematoma 2)** | | | | |
| --- | --- | --- | --- | --- |
|  | **Excellent outcome (mRS 0-1)** | | **Functional independence (mRS 0-2)** | |
|  | **Odds ratio (95% CI)** | ***P-*value** | **Odds ratio (95% CI)** | ***P-*value** |
| Age, years | 0.99 (0.97-1.01) | 0.238 | 0.98 (0.90-1.00) | 0.080 |
| Baseline NIHSS | 0.95 (0.90-1.00) | 0.044 | 0.95 (0.90-1.00) | 0.043 |
| MCA occlusion | 0.66 (0.39-1.13) | 0.133 | 0.49 (0.29-0.88) | 0.025 |
| Baseline Ischemic core volume, mL | 0.96 (0.90-1.02) | 0.209 | 0.98 (0.92-1.04) | 0.484 |
| Successful reperfusion | 2.45 (1.46-4.20) | <0.001 | 2.18 (1.33-3.59) | 0.002 |
| sICH | 0.13 (0.03-0.39) | 0.001 | 0.22 (0.09-0.49) | <0.001 |
| CI refers to confidence interval; FIV, follow-up infarct volume; MCA, middle cerebral artery; mRS, modified Rankin Scale; NIHSS, National Institutes of Health Stroke Score; PSI, penumbra salvage index; sICH, symptomatic intracerebral hemorrhage. | | | | |

| **Supplementary Table 3: Multivariate linear regression analysis for the subgroup patients with complete reperfusion** | | | | |
| --- | --- | --- | --- | --- |
|  | **Penumbra salvage index**  **Coefficient (95% CI)** | ***P* Value** | **Follow-up infarct volume**  **Coefficient (95% CI)** | ***P* Value** |
| Age | -0.01 (-0.01, 0.01) | 0.871 | -0.09 (-0.23, 0.04) | 0.162 |
| Baseline NIHSS | -0.01 (-0.01, 0.01) | 0.296 | -0.04 (-0.38, 0.30) | 0.823 |
| MCA occlusion | 0.02 (-0.03, 0.07) | 0.389 | -1.00 (-4.47, 2.47) | 0.571 |
| Baseline Ischemic core volume | -0.01 (-0.01, 0.01) | 0.799 | 0.48 (0.08, 0.89) | 0.019 |
| Complete reperfusion | 0.12 (0.07, 0.17) | <0.001 | -4.16 (-7.35, -0.99) | 0.010 |
| CI refers to confidence interval; MCA, middle cerebral artery; | | | | |

| **Supplementary Table 4: Outcome model multivariate logistic regression analysis for the subgroup patients with complete reperfusion** | | | | |
| --- | --- | --- | --- | --- |
|  | **Excellent outcome (mRS 0-1)** | | **Functional independence (mRS 0-2)** | |
|  | **Odds ratio (95% CI)** | ***P-*value** | **Odds ratio (95% CI)** | ***P-*value** |
| Age, years | 0.98 (0.95-1.00) | 0.104 | 0.97 (0.94-0.99) | 0.014 |
| Baseline NIHSS | 0.93 (0.87-0.99) | 0.035 | 0.96 (0.90-1.02) | 0.177 |
| MCA occlusion | 0.63 (0.32-1.23) | 0.176 | 0.42 (0.20-0.85) | 0.020 |
| Baseline Ischemic core volume, mL | 0.95 (0.88-1.03) | 0.342 | 0.96 (0.88-1.03) | 0.245 |
| Complete reperfusion | 1.31 (0.70-2.46) | 0.400 | 1.66 (0.89-3.09) | 0.111 |
| Penumbra salvage index, per 10% | 1.38 (1.12-1.75) | 0.004 | 1.08 (0.89-1.32) | 0.440 |
| Follow-up infarct volume, mL | 0.98 (0.95-1.01) | 0.215 | 0.98 (0.95-1.01) | 0.167 |
| CI refers to confidence interval; FIV, follow-up infarct volume; MCA, middle cerebral artery; mRS, modified Rankin Scale; NIHSS, National Institutes of Health Stroke Score; PSI, penumbra salvage index. | | | | |
